# Human Adenosine A_2A_ Receptor: Molecular Mechanism of Ligand Binding and Activation

**DOI:** 10.3389/fphar.2017.00898

**Published:** 2017-12-14

**Authors:** Byron Carpenter, Guillaume Lebon

**Affiliations:** ^1^Warwick Integrative Synthetic Biology Centre, School of Life Sciences, University of Warwick, Coventry, United Kingdom; ^2^Institut de Génomique Fonctionnelle, Neuroscience Department, UMR CNRS 5203, INSERM U1191, Université de Montpellier, Montpellier, France

**Keywords:** GPCR, adenosine, structural biology, G protein, drugs, x-ray diffraction

## Abstract

Adenosine receptors (ARs) comprise the P1 class of purinergic receptors and belong to the largest family of integral membrane proteins in the human genome, the G protein-coupled receptors (GPCRs). ARs are classified into four subtypes, A_1_, A_2A_, A_2B_, and A_3_, which are all activated by extracellular adenosine, and play central roles in a broad range of physiological processes, including sleep regulation, angiogenesis and modulation of the immune system. ARs are potential therapeutic targets in a variety of pathophysiological conditions, including sleep disorders, cancer, and dementia, which has made them important targets for structural biology. Over a decade of research and innovation has culminated with the publication of more than 30 crystal structures of the human adenosine A_2A_ receptor (A_2A_R), making it one of the best structurally characterized GPCRs at the atomic level. In this review we analyze the structural data reported for A_2A_R that described for the first time the binding of mode of antagonists, including newly developed drug candidates, synthetic and endogenous agonists, sodium ions and an engineered G protein. These structures have revealed the key conformational changes induced upon agonist and G protein binding that are central to signal transduction by A_2A_R, and have highlighted both similarities and differences in the activation mechanism of this receptor compared to other class A GPCRs. Finally, comparison of A_2A_R with the recently solved structures of A_1_R has provided the first structural insight into the molecular determinants of ligand binding specificity in different AR subtypes.

**Key Concepts**

1. **A_2A_R crystallization: selection of different conformational states**
  Structure determination of A_2A_R required the application of novel protein engineering techniques to lock the receptor in defined conformational states and facilitate the growth of crystals that diffract to high resolution.
2. **Structural determinants of A_2A_R ligand binding and selectivity**
  The atomic resolution structural features of A_2A_R that dictate which ligands it can bind and whether the ligands act as agonists, to promote signaling, or antagonists, to block signaling.
3. **Ligand-induced activation of A_2A_R**
  The molecular changes that are induced in A_2A_R by agonist binding, which facilitate G protein coupling and ultimately signal transduction.
4. **Structural diversity of the adenosine receptor family**
  The difference in the primary and ternary structure between the four AR subtypes that is ultimately responsible for their ligand-binding specificity and pharmacological profiles.

## Introduction

Purinergic signaling is predominantly mediated by extracellular purine nucleosides and nucleotides, including adenosine and adenosine triphosphate (ATP), but also by purine bases such as caffeine and xanthine. Purinergic receptors are integral membrane protein that are divided into three classes, P1 (better known as adenosine receptors), P2Y, and P2X (Burnstock, 2006). Both P1 and P2Y receptors belong to the G protein-coupled receptor (GPCR) family, whereas P2X receptors are ATP-gated ion channels. Adenosine receptors (ARs) are divided into four subtypes, A_1_, A_2A_, A_2B_, and A_3_ (Fredholm et al., 2001), which are broadly expressed in the central nervous system as well as peripheral tissues of the cardiovascular, respiratory, renal, and immune systems (Fredholm et al., 2001, 2011). Extracellular adenosine is the endogenous agonist for all ARs, however differences in the adenosine binding affinity, tissue distribution, expression level and G protein coupling preference between the subtypes gives each a distinct signaling profile (Cieslak et al., 2008; Fredholm, 2014). A_1_R and A_3_R predominantly activate heterotrimeric G proteins belonging to the Gα_i/o_ family, which inhibit cAMP production by adenylate cyclase, in contrast A_2A_R and A_2B_R predominantly activate Gα_s_ family members, which stimulate cAMP production (Jacobson and Gao, 2006). G protein βγ subunits also contribute to signaling through the mitogen-activated protein kinase (MAPK) and phospholipase C (PLC) pathways (Jacobson and Gao, 2006). ARs mediate the general cytoprotective functions associated with extracellular adenosine, with some of the key physiological processes regulated by individual subtypes being: sleep, vasoconstriction and inhibition of neurotransmitter release by A_1_R; sleep, angiogenesis, and immunosuppression by A_2A_R; vascular integrity and myocardial preconditioning by A_2B_R; mast cell regulation and myocardial preconditioning by A_3_R (Fredholm et al., 2011).

ARs have been proposed as potential targets in a wide variety of pathophysiological conditions, including arrhythmia, ischemia, sleep disorders, pain, dementia, Parkinson's, renal failure, asthma, type 2 diabetes, glaucoma, inflammation, and cancer (Jacobson and Gao, 2006; Cieslak et al., 2008; Sawynok, 2016). However, one of the challenges of therapeutic intervention has been targeting individual AR subtypes with sufficient specificity to limit off-target side effects (Chen et al., 2013). Medicinal chemistry approaches have been used to develop an array of compounds that exhibit improved subtype specificity (Müller and Jacobson, 2011), but very few have been approved for clinical use, due in part to the persistence of undesirable side effects (Chen et al., 2013; Glukhova et al., 2017). Further improvements in subtype specificity, coupled with the development of allosteric modulators (Gentry et al., 2015) that bind outside the orthosteric site, and biased ligands (Kenakin and Christopoulos, 2013) that can target a distinct signaling pathway associated with an individual AR subtype, may help to eliminate side effects entirely. Structure-based drug design, which involves *in silico* screening of vast compound libraries against experimentally determined receptor structures, offers huge potential for the development of a new generation of highly selective orthosteric, allosteric, and biased ligands, however, the difficulty of crystallizing GPCRs has, until recently, hindered this approach (Jazayeri et al., 2015).

Structure determination of GPCRs is notoriously challenging due to their conformationally dynamic nature and poor thermostability when extracted from the plasma membrane. During the past decade crystallization strategies have been developed that have revolutionized the structural biology of GPCRs, these include protein engineering approaches, such as fusion proteins (Cherezov et al., 2007; Chun et al., 2012), antibodies (Rasmussen et al., 2007, 2011a) and conformational thermostabilization (Magnani et al., 2008; Serrano-Vega et al., 2008; Shibata et al., 2009), as well as technical developments, such as the lipidic cubic phase (LCP) (Landau and Rosenbusch, 1996; Caffrey, 2015). Human A_2A_R has been at the forefront of this revolution and is now one of the best structurally characterized GPCRs, with more than 30 structures deposited in the protein data bank (PDB; Table 1). It is the only receptor for which structures of three distinct activation states have been reported, namely the inactive conformation bound to an antagonist or inverse agonist (Jaakola et al., 2008), an intermediate-active conformation bound to an agonist (Lebon et al., 2011b; Xu et al., 2011), and the active conformation bound to both an agonist and engineered G protein (Carpenter et al., 2016). Crystallization of the other AR subtypes has proved more difficult and it is only during the past year that structures of A_1_R have been published (Cheng et al., 2017; Glukhova et al., 2017). Significantly, these have provided the first atomic resolution insight in to the molecular determinants of ligand binding specificity in different AR subtypes.

**Table 1:** Adenosine receptor X-ray crystal structures.

**Receptor subtype**	**Conformational state**	**Ligand class**	**Ligand name**	**Fusion protein**	**Thermostabilized**	**Binding partner**	**Resolution (Å)**	**PDB code**	**References**
A_1_	Inactive	Antagonist	DU172^a^	BRIL	No	None	3.2	5UEN	Glukhova et al., 2017
	Inactive	Antagonist	PSB36	BRIL	Yes	None	3.3	5N2S	Cheng et al., 2017
A_2A_	Inactive	Inverse agonist	ZM241385	T4L	No	None	2.6	3EML	Jaakola et al., 2008
	Inactive	Inverse agonist	ZM241385	None	Yes	None	3.3	3PWH	Doré et al., 2011
	Inactive	Antagonist	XAC	None	Yes	None	3.3	3REY	Doré et al., 2011
	Inactive	Antagonist	Caffeine	None	Yes	None	3.6	3RFM	Doré et al., 2011
	Inactive	Inverse agonist	ZM241385	BRIL	No	None	1.8	4EIY	Liu et al., 2012
	Inactive	Antagonist	T4G^b^	None	Yes	None	3.3	3UZA	Congreve et al., 2012
	Inactive	Antagonist	T4E^b^	None	Yes	None	3.3	3UZC	Congreve et al., 2012
	Inactive	Inverse agonist	ZM241385	None	No	Fab2823	2.7	3VG9	Hino et al., 2012
	Inactive	Inverse agonist	ZM241385	None	No	Fab2823	3.1	3VGA	Hino et al., 2012
	Inactive	Inverse agonist	ZM241385	BRIL	Yes	None	1.7	5IU4	Segala et al., 2016
	Inactive	Antagonist	6DY^b^	BRIL	Yes	None	1.9	5IU7	Segala et al., 2016
	Inactive	Antagonist	6DZ^b^	BRIL	Yes	None	2.0	5IU8	Segala et al., 2016
	Inactive	Antagonist	6DX^b^	BRIL	Yes	None	2.2	5IUA	Segala et al., 2016
	Inactive	Antagonist	6DV^b^	BRIL	Yes	None	2.1	5IUB	Segala et al., 2016
	Inactive	Inverse agonist	ZM241385	BRIL	No	None	2.5	5K2A	Batyuk et al., 2016
	Inactive	Inverse agonist	ZM241385	BRIL	No	None	2.5	5K2B	Batyuk et al., 2016
	Inactive	Inverse agonist	ZM241385	BRIL	No	None	1.9	5K2C	Batyuk et al., 2016
	Inactive	Inverse agonist	ZM241385	BRIL	No	None	1.9	5K2D	Batyuk et al., 2016
	Inactive	Inverse agonist	ZM241385	BRIL	No	None	2.8	5JTB	Melnikov et al., 2017
	Inactive	Antagonist	8D1^b^	BRIL	No	None	3.5	5UIG	Sun et al., 2017
	Inactive	Inverse agonist	ZM241385	BRIL	No	None	3.2	5UVI	Martin-Garcia et al., 2017
	Inactive	Antagonist	Caffeine	BRIL	Yes	None	2.1	5MZP	Cheng et al., 2017
	Inactive	Antagonist	Theophylline	BRIL	Yes	None	2.0	5MZJ	Cheng et al., 2017
	Inactive	Antagonist	PSB36	BRIL	Yes	None	2.8	5N2R	Cheng et al., 2017
	Inactive	Inverse agonist	ZM241385	BRIL	Yes	None	2.1	5NLX	Weinert et al., 2017
	Inactive	Inverse agonist	ZM241385	BRIL	Yes	None	2.0	5NM2	Weinert et al., 2017
	Inactive	Inverse agonist	ZM241385	BRIL	Yes	None	1.7	5NM4	Weinert et al., 2017
	Intermediate-active	Agonist	UK-432097	T4L	No	None	2.7	3QAK	Xu et al., 2011
	Intermediate-active	Agonist	Adenosine	None	Yes	None	3.0	2YDO	Lebon et al., 2011b
	Intermediate-active	Agonist	NECA	None	Yes	None	2.6	2YDV	Lebon et al., 2011b
	Intermediate-active	Agonist	CGS21680	None	Yes	None	2.6	4UG2	Lebon et al., 2015
	Intermediate-active	Agonist	CGS21680	None	Yes	None	2.6	4UHR	Lebon et al., 2015
	Active	Agonist	NECA	None	No	Mini-G_s_	3.4	5G53	Carpenter et al., 2016

a *Covalently bound antagonist*.

b *Ligand nomenclature as used in the PDB*.

In this review we consolidate and analyze all of the structural information published during the past decade, which provides a near complete picture of A_2A_R activation. We compare the binding mode of antagonists, including the widely consumed stimulant caffeine (Doré et al., 2011; Cheng et al., 2017), and agonists, including the endogenous ligand adenosine (Lebon et al., 2011b). We highlight the agonist-induced conformational changes that activate A_2A_R (Lebon et al., 2011b; Xu et al., 2011), and the cooperative conformational changes induced by G protein coupling (Carpenter et al., 2016). Finally, we compare A_2A_R with the recently solved structures of A_1_R (Cheng et al., 2017; Glukhova et al., 2017) and discuss the current evidence for the molecular basis of ligand binding specificity in different AR subtypes.

## A_2A_R crystallization: selection of different conformational states

GPCRs are challenging targets for structural studies for three main reasons. First, flexibility and conformationally dynamics play a central role in receptor activation by maintaining a dynamic equilibrium between different conformational states (Kobilka and Deupi, 2007). Ligand binding is often insufficient to trap the receptor in a distinct conformation (Manglik et al., 2015; Ye et al., 2016), which can perturb the growth of crystals that diffract to high resolution (Cherezov et al., 2007; Warne et al., 2008; Tate and Schertler, 2009). Second, GPCRs are highly unstable upon extraction from the membrane by detergent solubilization, which makes purification of the receptors both technically challenging and inefficient (Serrano-Vega et al., 2008). Third, class A receptors are compact proteins that typically have only minimal hydrophilic surface area capable of forming crystal contacts. Structure determination of virtually all GPCRs, including A_2A_R, has therefore required the development of novel protein engineering strategies (discussed below), crystallization techniques, including LCP (Landau and Rosenbusch, 1996; Xu et al., 2011; Caffrey, 2015), and data collection methods, including the use of micrometer-sized X-ray beams (Moukhametzianov et al., 2008) or serial crystallography (Weinert et al., 2017), in order to obtain well-diffracting crystals and collect high resolution diffraction data.

**Figure 1:**
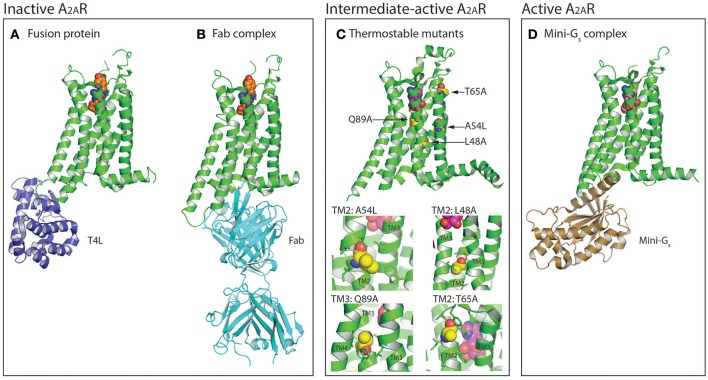
Protein engineering strategies used for the selection and crystallization of distinct A_2A_R conformations. **(A)** Crystal structure of A_2A_R (colored green) in the inactive conformation bound to the inverse agonist ZM241385 (shown as spheres, carbon atoms colored orange) with T4 lysozyme (T4L; colored blue) fused between the intracellular ends of H5 and H6 (PDB: 3EML) (Jaakola et al., 2008). The T4L fusion protein has also been substituted with apocytochrome b_562_RIL (BRIL; not shown) (Liu et al., 2012). **(B)** Structure of A_2A_R in the inactive conformation bound to ZM241385 (shown as spheres, carbon atoms colored orange) and an antibody F_ab_ fragment (colored cyan) that acts as an intracellular inverse agonist (PDB: 3VG9 and 3VGA) (Hino et al., 2012). **(C)** Crystal structure of thermostabilized A_2A_R-GL26 in the intermediate-active conformation bound to the agonist adenosine (shown as spheres, carbon atoms colored magenta), the four thermostabilizing mutations (L48A^2.46^, A54L^2.52^, T65A^2.63^, and Q89A^3.37^) are shown as spheres and their carbon atoms colored yellow (PDB: 2YDO) (Lebon et al., 2011b). In the expanded view the residues with which the thermostabilizing mutations interact (distance <4 Å) are shown as sticks. Conformational thermostabilization has facilitated the crystallization of A_2A_R in both the agonist- and antagonist- bound conformations. **(D)** Structure of A_2A_R in the active conformation bound to NECA (shown as spheres, carbon atoms colored light pink) and an engineered G protein mini-G_s_ (colored brown; PDB: 5G53) (Carpenter et al., 2016). Figures were prepared using PyMOL (PyMOL™ Molecular Graphics System, Version 1.8.6.0).

The first structure of A_2A_R was solved bound to the inverse agonist ZM241385 at 2.6 Å resolution (Jaakola et al., 2008). This structure was facilitated by a combined approach of using a high affinity ligand, which locks the receptor in its inactive state, and by replacing the third intracellular loop (ICL3) with a T4 lysozyme (T4L) fusion protein (Rosenbaum et al., 2007), which increases the hydrophilic surface area available for crystal contact formation (Figure 1A). This fusion protein strategy was subsequently modified to utilize apocytochrome b_562_RIL (BRIL) instead of T4L (Liu et al., 2012), which resulted in the solution of seven additional structures of A_2A_R bound to ZM241385 ranging in resolution from 3.2 to 1.8 Å (Liu et al., 2012; Batyuk et al., 2016; Martin-Garcia et al., 2017; Melnikov et al., 2017), and one structure bound to the antagonist 8D1 (Sun et al., 2017) (Table 1). Conformational thermostabilization, which utilizes alanine scanning mutagenesis to identify point mutations that stabilize the receptor in a particular conformational state and increase its thermostability in detergent (Magnani et al., 2008, 2016), was also applied to solve the structure of A_2A_R bound to ZM241385 (Doré et al., 2011). The construct A_2A_R-StaR2 contained eight thermostabilizing mutations (A54L^2.52^, T88A^3.36^, R107A^3.55^, K122^4.43^, L202A^5.63^, L235A^6.37^, V239A^6.41^, and S277A^7.42^; superscripts refer to Ballesteros–Weinstein numbering) (Ballesteros and Weinstein, 1995) that increased the stability of the receptor in the detergent dodecylmaltoside (DDM) by ~18°C. A_2A_R-StaR2 has since been crystallized bound to four different antagonists XAC, caffeine, T4G and T4E (Doré et al., 2011; Congreve et al., 2012). Conformational thermostabilization has also been used in combination with a BRIL fusion protein to facilitate the crystallization of A_2A_R bound to the antagonists 6DY, 6DZ, 6DX, 6DV, ZM241385, caffeine, theophylline, and PSB36 (Segala et al., 2016; Cheng et al., 2017). Furthermore, ZM241385-bound A_2A_R has been co-crystallized in complex with an antibody F_ab_ fragment (Fab2823), which acts as an intracellular inverse agonist locking the receptor in its inactive conformation, and also helps to increase the hydrophilic protein surface available for crystal contact formation (Figure 1B) (Hino et al., 2012).

The first agonist bound structure of A_2A_R was solved in complex with the synthetic agonist UK-432097, using the T4L fusion strategy (Xu et al., 2011). This ligand is large, approximately three times the molecular weight of adenosine, and imparts a significant increase in thermostability to the receptor. Crystallization of A_2A_R bound to smaller less stabilizing agonists required the application of the conformational thermostabilization methodology (Figure 1C). In this case the receptor was thermostabilized in the presence of the agonist NECA, and four mutations (L48A^2.46^, A54L^2.52^, T65A^2.63^, and Q89A^3.37^) were combined in the final construct (A_2A_R-GL26) (Lebon et al., 2011a). The stability of A_2A_R-GL26 was ~16°C higher than the wild type receptor in DDM, which facilitated structure determination of the receptor in complex with the small agonists adenosine and NECA (Lebon et al., 2011b), as well as the larger CGS21680 (Lebon et al., 2015). All four agonist-bound structures adopted a conformation that was different from the inactive state, but that did not fully resemble the active state of the β_2_ adrenergic receptor (β_2_AR) in complex with heterotrimeric G_s_ (Rasmussen et al., 2011b), this conformation was therefore defined as the intermediate-active state (Lebon et al., 2011b). Interestingly, the high affinity agonist UK-432097 was sufficiently stabilizing to allow the crystallization of non-thermostabilized A_2A_R in the intermediate-active state. This is likely due to the large size of the ligand, which results in it forming more extensive molecular contacts with the receptor, in particular UK-432097 forms four additional direct hydrogen bonds with A_2A_R compared to adenosine. In the case of small low affinity agonists, such as adenosine, conformational thermostabilization was absolutely necessary to facilitate structured determination.

The agonist-bound A_2A_R structures exhibited some characteristics of the active receptor (Lebon et al., 2012), however stabilizing the fully active conformation requires simultaneous binding of the agonist and G protein or a functional mimetic (Rasmussen et al., 2011a,b). Co-crystallization of GPCRs in complex with heterotrimeric G proteins is the ideal case for characterizing receptors in their active state, however this approach is challenging, due in part to the large size and conformationally dynamic nature of the G protein (Westfield et al., 2011). The development of nanobodies that act as surrogates of heterotrimeric G proteins has proved to be a powerful approach to crystallize receptors in their active conformation (Steyaert and Kobilka, 2011), however the disadvantage of this method is that nanobodies do not recapitulate the native GPCR–G protein interface. Structure determination of A_2A_R in its active conformation was achieved using a recently developed minimal G protein, which is composed of a single engineered domain from the Gα_s_ subunit (Figure 1D) (Carpenter and Tate, 2016, 2017b,c). This mini G protein (mini-G_s_) sufficiently stabilized NECA-bound A_2A_R in its fully active conformation to facilitate crystallization and structure determination at 3.4 Å resolution (Carpenter et al., 2016). This approach has now been applied to most heterotrimeric G proteins (Nehmé et al., 2017) and should play an important role solving high-resolution structures of other GPCRs in their active state (Strege et al., 2017).

## Structural determinants of A_2A_R ligand binding and selectivity

**Figure 2:**
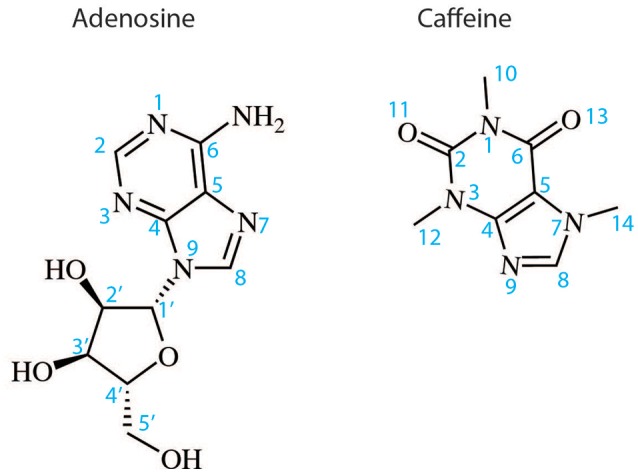
Two dimensional structures of the endogenous A_2A_R agonist adenosine and the naturally occurring antagonist caffeine. The atom numbering indicated is used in the text to describe the binding of these ligands and their derivatives to A_2A_R.

**Figure 3:**
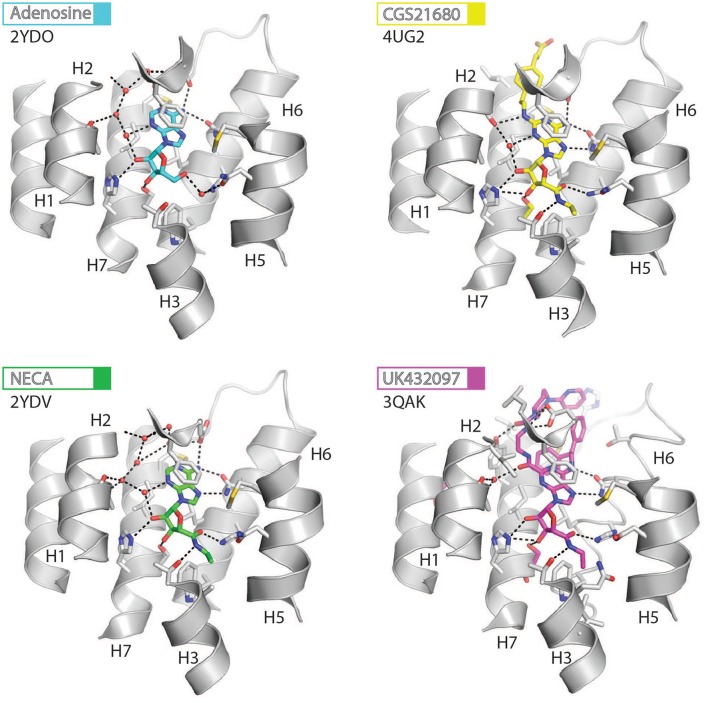
High-resolution view of the agonist-binding site of human A_2A_R. Four different agonists have been co-crystallized with A_2A_R in the intermediate-active conformation. The receptor is shown as cartoons and colored gray, residues and side chains that interact with the ligand are shown as sticks and colored by element (carbon, gray; nitrogen, blue; oxygen, red; sulfur, yellow). The ligands are shown as sticks and their carbon atoms are colored to match their labels, PDB codes are shown in the figure. Polar contacts are represented as dashed lines and water molecules are shown as red spheres.

**Figure 4:**
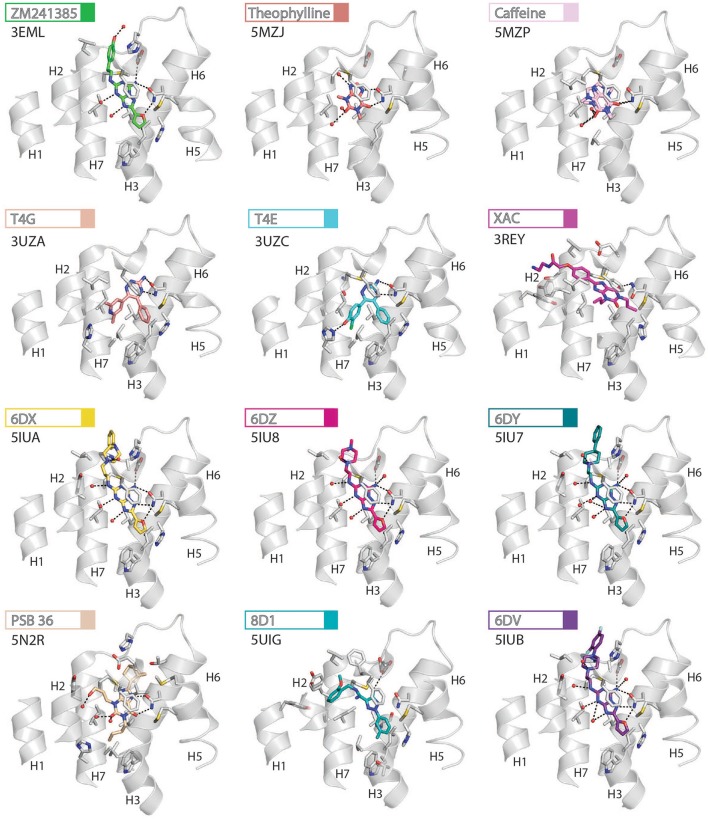
High-resolution view of the antagonist- or inverse agonist- binding site of human A_2A_R. Twelve different antagonists or inverse agonists have been co-crystallized with A_2A_R, in the inactive conformation. The receptor is shown as cartoons and colored gray, residues and side chains that interact with the ligand are shown as sticks and colored by element (carbon, gray; nitrogen, blue; oxygen, red; sulfur, yellow). The ligands are shown as sticks and their carbon atoms are colored to match their labels, PDB codes are shown in the figure. Polar contacts are represented as dashed lines and water molecules are shown as red spheres. Note that for caffeine the two distinct binding orientations that were observed in the structure are overlaid.

**Figure 5:**
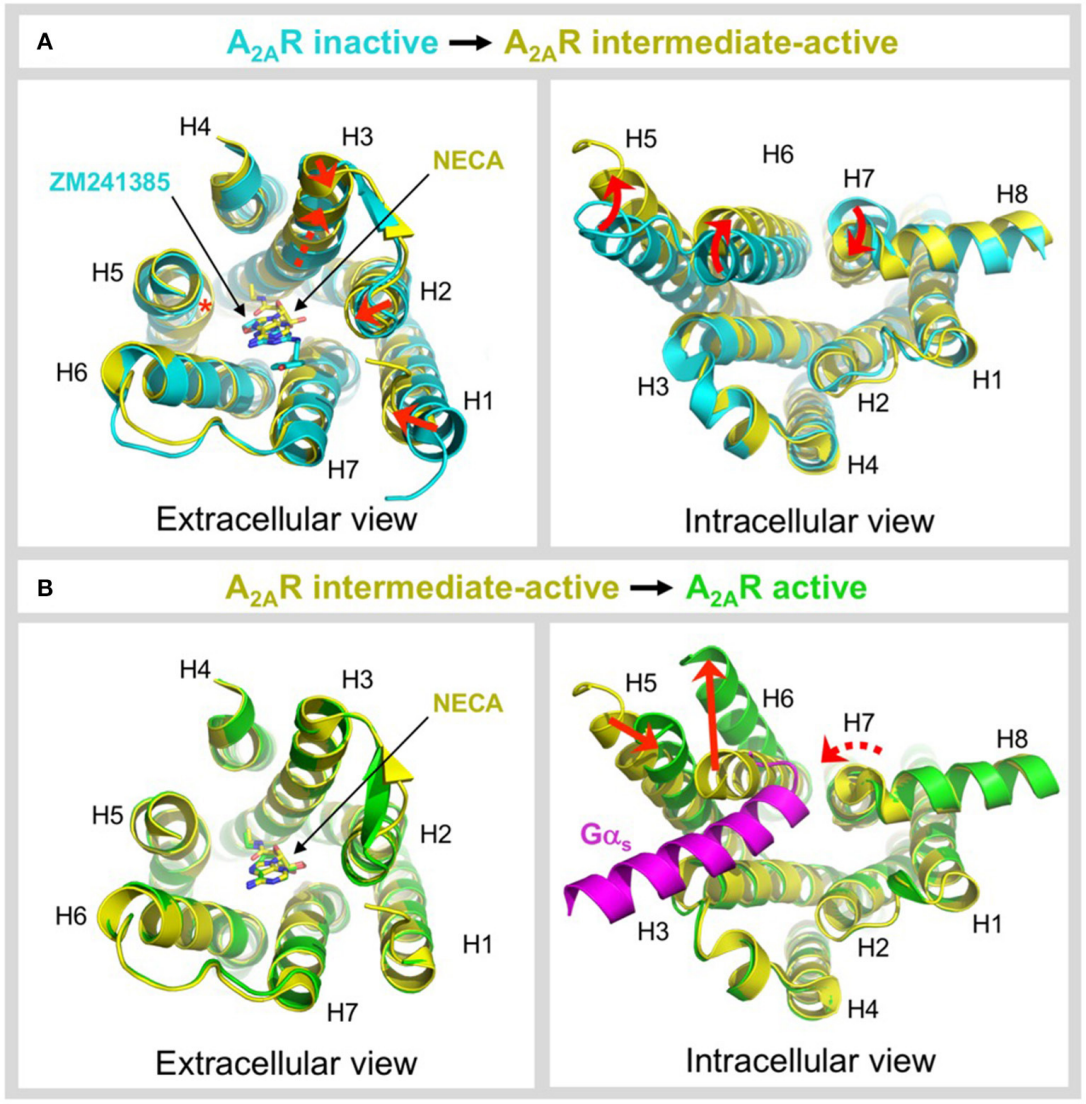
Ligand-induced activation of A_2A_R. **(A)** Conformational changes associated with agonist-induced transition from the inactive state (colored cyan) to the intermediate-active state (colored yellow; PDB: 2YDV) (Lebon et al., 2011b). Two different inactive state structures were used for the alignments, the extracellular view uses 4EIY (Liu et al., 2012) because it is the highest resolution non-thermostabilized structure available, the intracellular view uses 3PWH (Doré et al., 2011) because it was crystallized without a fusion protein in ICL3. The extracellular view shows the 1–2 Å inward shifts in the ends of H1, H2, and H3 (indicated by a red arrows), the 2 Å translocation of H3 along its axis (indicated by a dashed red arrow), and the inward bulge in H5 (indicated by a red asterisk). The inverse agonist ZM241385 (colored cyan) and agonist NECA (colored yellow) are shown as sticks. The intracellular view shows the combined rotational and lateral movements in H5, H6, and H7 (indicated by curved red arrows). H5 and H6 are displaced outwards by 7 and 5 Å, respectively, while H7 moves inwards in by 4 Å. **(B)** Conformational changes associated with G protein-induced transition from the intermediate-active state (colored yellow; PDB: 2YDV) (Lebon et al., 2011b) to the active state (colored green; PDB: 5G53) (Carpenter et al., 2016). No significant changes are observed on the extracellular side of the receptor, and the position of NECA (shown as sticks) is essentially identical in both states (Carpenter et al., 2016). On the intracellular side of the receptor an outward movement of H6 by 14 Å (indicated by a red arrow) is required to accommodate binding of mini-G_s_ (colored magenta). H5 moves inwards by 5 Å (indicated by a red arrow) and forms direct contacts the α5 helix of mini-G_s_. H7 undergoes a rotation, without significant lateral movement (indicated by a curved and dashed red arrow), which reorients the H7-H8 boundary to interact with the C-terminus of mini-G_s_ (Carpenter et al., 2016). Note that for clarity, ECL2 has been omitted from the extracellular view of all alignments.

The orthosteric binding site of A_2A_R can be defined by the residues involved in binding the endogenous agonist adenosine and the naturally occurring antagonist caffeine (see Figure 2 for the ligand structures and atom numbering, and Table 2 for A_2A_R-ligand contacts). Adenosine and caffeine share a common xanthine moiety that in both cases establishes van der Waals interactions with M177^5.38^, M270^7.35^ and I274^7.39^, and a π-stacking interaction with the aromatic ring of F168, which is part of the helical portion of extracellular loop 2 (ECL2; Figures 3, 4) (Doré et al., 2011; Lebon et al., 2011b; Cheng et al., 2017). N253^6.55^ forms hydrogen bonds with either the amine groups at positions C6 and N7 of the adenine moiety of adenosine (Lebon et al., 2011b) or O11/O13 from the xanthine heterocycle of caffeine, for which two distinct binding orientations have been observed (Cheng et al., 2017). The ribose moiety of adenosine forms van der Waals interactions with V84^3.32^, L85^3.33^, T88^3.36^, W246^6.48^, and L249^6.51^; these residues form similar contacts with many antagonist. The main difference between the adenosine- and caffeine-binding modes is the formation of hydrogen bonds from the hydroxyl groups at positions C2 and C3 of the ribose moiety of adenosine to S277^7.42^ and H278^7.43^. The recent high-resolution structure of A_2A_R bound to caffeine (2.10 Å) did reveal a water-mediated contact between H278^7.43^ and O11 of caffeine (Cheng et al., 2017). However, the presence of this water molecule highlights the difference in the distance between these atoms compared to the adenosine-bound state, where a direct interaction is observed. This distance is reduced concomitantly with the conformational changes in H3 and H7 associated with agonist binding to the receptor (Figure 5) (Lebon et al., 2012). All agonists that have been co-crystallized with A_2A_R engage H3 through T88^3.36^ and H7 through S277^7.42^/H278^7.43^ (Table 2). Some antagonists do interact with either T88^3.36^ or S277^7.42^/H278^7.43^, but never at the same time, which suggests that the simultaneous engagement of these residues in H3 and H7 may be a key determinant of agonist activity.

**Table 2:** A_2A_R-ligand interactions.

**Secondary structure element**	**A_2A_R residue**	**Ligand (agonist/antagonist)**															
		**Adenosine(2YDO)**	**NECA(2YDV)**	**CGS21680 (4UG2)**	**UK-432097 (3QAK)**	**Caffeine (5MZP)**	**Theophylline (5MZJ)**	**ZM241385 (3EML)**	**T4G (3UZA)**	**T4E (3UZC)**	**XAC (3REY)**	**6DX (5IUA)**	**6DZ (5IU8)**	**6DY (5IU7)**	**6DV (5IUB)**	**PSB36 (5N2R)**	**8D1 (5UIG)**
H1	Y9																
H2	A63																
	I66																
	S67																
H3	A81																
	V84																
	L85																
	T/A88	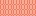	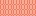	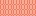	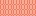												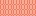
	Q/A89																
	I92																
ECL2	L167																
	F168																
	E169																
	M174																
H5	M177																
	N181																
H6	W246																
	L249																
	H250																
	I252																
	N253																
	T256																
ECL3	H264																
	A265																
H7	P266																
	L267																
	M270																
	Y271																
	I274																
	S/A277																
	H278																

*A_2A_R residues that directly interact with each ligand (distances < 4 Å) are indicated by filled cells and colored according to the ligand type with which they interact (agonists only, red; antagonists only, blue; both agonists and antagonists, gray). Residues F168^ECL2^, M177^5.38^, L249^6.51^, N253^6.55^ and I274^7.39^ interact with all agonists and antagonists for which structures have been solved and are indicated by filled cells colored green. T88^3.36^ interacts with all agonists for which structures have been solved and is a key residue in agonist-induced receptor activation, however since it also interacts with the antagonist 8D1 (discussed in the text) it is indicated by hatched cells colored red. The secondary structure element in which each residue is located is shown in the left hand column (H, transmembrane helix; ECL, extracellular loop). The PDB codes of the A_2A_R structures used for analysis are shown in parentheses next to each ligand. Note that several of the A_2A_R structures contain thermostabilizing mutations, including T88A/S277A (caffeine, theophylline, T4G, T4E, XAC, 6DX, 6DZ, 6DY, 6DV, PSB36) and Q89A (adenosine, NECA and CGS21680)*.

Structures of A_2A_R have been solved in complex with three high-affinity synthetic agonists NECA, CGS21680 and UK-432097 (Lebon et al., 2011b, 2015; Xu et al., 2011). These agonists share a core adenosine moiety, the binding mode of which is very similar to adenosine itself, with the ribose group establishing hydrogen bonds with S277^7.42^ and H278^7.43^. For all three ligands the N-ethylcarboxyamido tail at position C5′ of the ribose ring extends deep into the binding pocket. The nitrogen and oxygen atoms form polar contacts with T88^3.36^ and H250^6.52^, respectively, and the substituent is further stabilized by van der Waals interactions with N181^5.42^, W246^6.48^ and to a lesser extent Q/A^3.37^. In contrast, the OH group at this position of adenosine interacts with N181^5.42^ and H250^6.52^ through water-mediated interactions and M177^5.38^ through a van der Waals interaction. The major difference between the binding mode of these agonists can be seen in the extracellular loops of the receptor. NECA and adenosine are both stabilized by a hydrogen bond with E169^ECL2^ (Lebon et al., 2011b); this residue also forms a salt bridge with H264^ECL3^, which closes the top of the binding pocket and is known to affect ligand binding kinetics (Guo et al., 2016; Segala et al., 2016). CGS21680 takes advantage of similar hydrogen bond between the amine group at position C6 of adenine moiety and E169^ECL2^ (Lebon et al., 2015). The larger (2-carboxyethyl)phenylethylamino substituent at the C2 position protrudes outside the binding pocket and is stabilized by van der Waals interactions with E169^ECL2^, H264^ECL3^, and L267^7.32^ as well as a hydrogen bond with S67^2.65^. As a consequence, the extracellular end of H2 is displaced inward compared to other agonist-bound structures, reducing the volume of the binding pocket. UK-432097 has two substituents on the adenine moiety, that make it an even larger molecule than CGS21680. The consequence is that the bulky 2-(3-[1-(pyridine-2-yl)piperidin-4-yl]ureido)ethylcarboxamido substitution at position C2 displaces ECL3 away from the binding pocket, which induces rotamer changes in E169^ECL2^ and H264^ECL3^ and breaks the salt bridge between these side chains (Xu et al., 2011). The urea group forms two hydrogen bonds with E169^ECL2^ as well as a hydrogen bond and van der Waals interactions with Y271^7.36^ on its opposite side. The pyridinyl-piperidine group, which extends furthest from the binding pocket, is stabilized by van der Waals interactions with L267^7.32^ and H264^ECL3^.

Like agonists, antagonists can exploit subsidiary binding sites by expanding their contact surface outside the orthosteric binding pocket. Starting with ZM241385, the trizolotriazine ring occupies the orthosteric binding site, and is surrounded by F168^ECL2^, L249^6.51^, M270^7.35^, I274^7.39^. Two residues, E169^ECL2^ and N253^6.55^, form hydrogen bonds with the amine group of the ZM241385 heterocycle and an additional hydrogen bond is established between N253^6.55^ and the oxygen of the furan ring. The formation of van der Waals interactions with M177^5.38^, W246^6.48^, L249^6.51^, and H250^6.52^ stabilize H5 and H6 against the furan ring. ZM241385 explores the chemical space outside the orthosteric site by taking advantage of the cavity on the extracellular surface of the receptor. Two distinct orientations have been observed for the phenol ring of ZM241385, in the first conformation the salt bridge between E169^ECL2^ and H264^ECL3^ is intact and the phenol ring forms van der Waals interactions with H264^ECL3^, L267^7.32^ and M270^7.35^ (Jaakola et al., 2008). Interestingly, the phenylethylamine group of ZM241385 adopts a binding mode similar to the (2-carboxyethyl)phenylethylamino substituent of CGS21680 (Lebon et al., 2015). The second conformation was observed in a thermostabilized receptor structure where the salt bridge between E169^ECL2^ and H264^ECL3^ is broken (Doré et al., 2011). In this case the phenol moiety is pointing toward H1 and H2 engaging S67^2.65^, Y271^7.36^, and I274^7.39^ through van der Waals interactions and A63^2.61^ through a hydrogen bond. Both Y9^1.35^ and Y271^7.36^ sidechains adopt different rotamers in order to accommodate the phenol substituent in this pose, demonstrating that this subsidiary pocket is conformationally malleable. The xanthine derivative XAC occupies the orthosteric pocket with the xanthine ring adopting a similar position to caffeine and the trizolotriazine heterocycle of ZM241385 (Doré et al., 2011). However, the xanthine core is branched at position N1 and N3; the N3 propyl substituent forms van der Waals interactions with I66^2.64^ and A81^3.29^ that are not observed for ZM241385. On the extracellular surface of the receptor the phenoxy-acetamide tail of XAC adopts a pose that is similar to the phenol group of ZM241385 in the thermostabilized receptor structure and is engaged by S67^2.65^, L267^7.32^, M270^7.35^, Y271^7.36^, and I274^7.39^. However, none of these interactions are unique to XAC since they are all observed for ZM241385 in either of its two poses. The recent structure of 8D1 (also known as compound-1) bound to A_2A_R provides another example of a ligand that exploits this subsidiary binding site (Sun et al., 2017). The methoxy phenyl substituent of 8D1 occupies the extracellular pocket formed by Y9^1.35^, A63^2.61^, I66^2.64^, S67^2.65^, L267^7.32^, M270^7.35^, Y271^7.36^, and I274^7.39^ in a similar way to XAC and ZM241385. In this case a unique interaction is observed between 8D1 and Y9^1.35^ that is not observed for any other ligand (Table 2). Thus, several different ligands and chemical substituents have been shown to take advantage of the subsidiary binding pocket located between H1, H2, and H7 on the extracellular surface of the receptor.

Cavities identified from high-resolution crystal structures provide valuable information for structure-based drug discovery. This can be best illustrated by the study of Congreve et al. who have reported the discovery of 1,2,4-triazine derivatives as A_2A_R antagonists by exploiting structural data (Congreve et al., 2012). The authors optimized a series of compounds and hypothesized that 1,2,4-triazine derivatives may occupy the same area of the binding pocket as the ribose moiety of agonists. Solution of the corresponding ligand–receptor structures showed that the triazine ring of T4E or T4G sits in a pocket similar to other ligands, and forms van der Waals interactions with F168^ECL2^ and two hydrogen bonds with N253^6.55^. The phenol substituent forms van der Waals interactions with L85^3.33^, M177^5.38^, W246^6.48^ L249^6.51^, and H250^6.52^. As predicted the dimethyl-pyridine group of T4G occupies the ribose pocket and the phenolic hydroxyl group of T4E establishes a hydrogen bond with H278^7.43^, but importantly the ligands retain their antagonistic properties.

Crystallographic studies have also highlighted the role that ECL2 and ECL3 play in ligand binding, specifically the effect of the salt bridge between E169^ECL2^ and H264^ECL3^ (Lebon et al., 2015; Guo et al., 2016; Segala et al., 2016). These two residue as well as the salt bridge that they form, stabilize several ligands in the binding pocket, as described above for ZM241385. The phenol ring of the ZM241385 has recently been replaced by a set of larger substitutions and their structures have been solved in complex with A_2A_R at high resolution (Segala et al., 2016). The substitutions were reported to affect the residence time of the ligands, with the ligand 6DV (also known as 12x) displaying the slowest off-rate. This observation is also in agreement with the agonist-bound structures where the salt bridge closes the binding site for adenosine, NECA and CGS21680 (Lebon et al., 2011b, 2015). Mutation of either E169^ECL2^ or H264^ECL3^ has been shown to impair the potency of NECA whereas only mutation of H264^ECL3^ affected CGS21680 (Lebon et al., 2015). The lower potency of agonists on these mutant receptors might be a consequence of a faster off-rate in absence of the salt bridge, however it appears that the stabilizing effect of the salt bridge can be compensated for by extended molecular contact formed by large molecules, such as CGS21680.

## Ligand-induced activation of A_2A_R

GPCRs exist in dynamic equilibrium between several discrete conformational states that are separated by energy barriers (Manglik et al., 2015; Ye et al., 2016). The inactive and active states are well-conserved between GPCRs (Rosenbaum et al., 2009; Carpenter and Tate, 2017a), however a number of intermediate conformations have been identified that appear to be more divergent (Lebon et al., 2011b; Xu et al., 2011; White et al., 2012; Manglik et al., 2015; Ye et al., 2016). Agonist binding to the receptor is one of the key events required to overcome the energy barrier of activation and increase occupancy of the conformational state(s) that are capable of binding heterotrimeric G proteins (Manglik et al., 2015; Ye et al., 2016; Prosser et al., 2017). A_2A_R is one of the only receptors for which an intermediate-active agonist-bound state has been crystallized (Lebon et al., 2011b; Xu et al., 2011). This structure has provided unique insight into the molecular changes that occur during two key activation events, namely agonist-induced transition from the inactive to intermediate-active state and G protein-induced transition from the intermediate-active to active state. The structures used for comparison are the inactive state bound to the inverse agonist ZM241385 (Doré et al., 2011; Liu et al., 2012), the intermediate-active state bound to the agonist NECA (Lebon et al., 2011b) and the active state bound to NECA and mini-G_s_ (Carpenter et al., 2016), see Figure 5. Agonist binding to A_2A_R triggers a series of conformational changes, most notably within the ligand-binding pocket and on the intracellular side of the receptor (Figure 5A). In the ligand binding pocket the most significant changes are a 2 Å translocation of H3 along its axis that is necessary to prevent steric clashes of V84^3.32^ and L85^3.33^ with NECA, the formation of contacts between the ribose moiety of the agonist and residues S277^7.42^ and H278^7.43^ in H7 that are completely absent in the inverse agonist-bound state, and an inward bulge in H5 that disrupts the local helix geometry and shifts C185^5.46^ toward the core of the receptor by 4 Å (Lebon et al., 2011b). Notably, the bulge in H5 has a knock-on effect on the position of H250^6.52^, which is shifted toward the ligand by 2 Å, a movement that would be sterically forbidden if the inverse agonist were bound (Lebon et al., 2011b, 2012; Xu et al., 2011). This bulge is also observed in other GPCR structures suggesting it is one of the key event in activation (Venkatakrishnan et al., 2013). Agonist binding causes only subtle conformational changes on the extracellular side of the receptor, including 1-2 Å inward shifts in the ends of H1, H2, and H3. More significant changes are observed on the intracellular side, which are thought to prime the receptor for G protein coupling (Venkatakrishnan et al., 2016), specifically combined rotations and lateral movements in the cytoplasmic ends of H5, H6, and H7, outwards by 7 and 5 Å for H5 and H6, respectively, and inwards by 4 Å for H7 (Figure 5A) (Lebon et al., 2011b; Xu et al., 2011). The ionic lock between R102^3.50^ and E228^6.30^, which is often engaged in inactive GPCR structures, is broken by the reorientation of H6.

Sodium ions (Na^+^) act as negative allosteric modulators of many class A GPCRs, typically stabilizing the ligand-free and antagonist-bound states, thereby imposing an energy barrier on receptor activation (Katritch et al., 2014). The high-resolution model of A_2A_R in its inactive state was the first GPCR structure to reveal the mode of Na^+^ binding (Liu et al., 2012). In A_2A_R the Na^+^-binding pocket is composed of residues D52^2.50^, S91^3.39^, T88^3.36^, W246^6.48^, N280^7.45^, and S281^7.46^; both D52^2.50^ and S91^3.39^ form direct polar interactions with Na^+^, whereas interactions with the other residues are mediated through a network of ordered water molecules (Liu et al., 2012; Gutiérrez-de-Terán et al., 2013). The conformational changes induced by agonist binding, particularly the rotation and inward shift of H7 and the outward shift of H6, cause the Na^+^-binding pocket to collapse, potentiating the displacement of Na^+^, thereby allowing the agonist to overcome the negative allosteric effect of Na^+^ and activate the receptor (Gutiérrez-de-Terán et al., 2013). Interestingly, the intermediate-active conformation is not observed in crystal structures of agonist-bound β_1_AR (Warne et al., 2011) or β_2_AR (Rosenbaum et al., 2011). Recent ^19^F nuclear magnetic resonance (^19^F NMR) studies have confirmed the existence of an additional active-like state in A_2A_R compared to β_2_AR (Manglik et al., 2015; Ye et al., 2016; Prosser et al., 2017), which may correlate with the intermediate-active conformation observed in the crystal structures, thus highlighting differences in the energy landscape of activation between these receptors.

In contrast to the widely distributed effects of agonist binding, G protein-induced conformational changes are confined to the intracellular side of the receptor (Figure 5B). G protein coupling has been reported to increase the agonist-binding affinity of A_2A_R between 10- and 40-fold (Murphree et al., 2002; Carpenter et al., 2016), yet it is striking that the conformation of the residues in the ligand-binding pocket and the position of the agonist are essentially identical in both the intermediate-active and active states (Carpenter et al., 2016). The most likely explanation is that in the intermediate-active structure the ligand-binding pocket has already adopted the high-affinity conformation (Carpenter et al., 2016). However, it cannot be discounted that the relatively small increase in the agonist-binding affinity of A_2A_R does not result from direct changes to the ligand-binding pocket, for example, a reduction in the conformational dynamics of the receptor caused by G protein binding could decrease the off-rate of the agonist, thus increasing its affinity (Carpenter and Tate, 2017a). On the intracellular side of the receptor the pivotal event is a 14 Å outward movement of the cytoplasmic end of H6 to accommodate binding of the α5 helix from mini-G_s_. The outward movement of H6 triggers both a 5 Å inward movement of H5, positioning it to interact with the α5 helix of the G protein, and a rotation within H7 that reorients the H7-H8 boundary to form extensive contacts with the C-terminus of mini-G_s_ (Carpenter et al., 2016). These helix movements also result in reorientation of R102^3.50^, Y197^5.58^, and Y288^7.53^ side chains within the core of the receptor, which likely stabilizes the active state, and which is one of the signatures of a GPCR in its active conformation (Carpenter and Tate, 2017a). It is clear that the intermediate-active state of A_2A_R is incompatible with G protein binding in its final orientation, i.e., that observed in the A_2A_R–mini-G_s_ structure (Carpenter et al., 2016), due to a large sterically forbidden clash between the α5 helix of mini-G_s_ and H6 of the receptor (Figure 5B). However, it is possible that the intermediate-active state of A_2A_R may be responsible for recognition of the G protein through an initial docking interaction, before cooperative conformational changes trigger nucleotide dissociation from the G protein and drive the receptor into its active conformation (Rasmussen et al., 2011b; Flock et al., 2015; Carpenter et al., 2016).

## Structural diversity of the adenosine receptor family

The amino acid sequence of the four AR subtypes is relatively poorly conserved, A_2A_R shares only 49, 56, and 39% identity with A_1_R, A_2B_R, and A_3_R, respectively (aligned over residues 1-312 of A_2A_R). This means that, despite there being a wealth of structural data available for A_2A_R, it has proved challenging to homology model other AR subtypes with sufficient accuracy for structure-based drug design applications (Glukhova et al., 2017). It is only during the past year that structures of an AR other than A_2A_R have been published, namely two structures of A_1_R bound to the xanthine antagonists DU172 (Glukhova et al., 2017) and PSB36 (Cheng et al., 2017). The two A_1_R structures are closely related and align with an RMSD of 0.6 Å (over 235 Cα atoms), they also align well with the ZM241385-bound structure of A_2A_R (Liu et al., 2012), with RMSDs of 0.8 Å (over 238 Cα atoms) and 1.0 Å (over 234 Cα atoms) for the DU172- and PSB36- bound structures, respectively. The intracellular side of both A_1_R structures strongly resemble the ZM241385-bound A_2A_R structure (3PWH), which was crystallized without a fusion protein in ICL3 (Doré et al., 2011), and the ionic lock between residues R105^3.50^ and E229^6.30^ is engaged in both cases (Cheng et al., 2017; Glukhova et al., 2017). The organization of the sodium-binding site is also well-conserved between A_1_R and A_2A_R, which supports mutagenesis data that indicated the negative allosteric effect of sodium on A_1_R was mediated through this site (Barbhaiya et al., 1996), although neither A_1_R structure was of sufficient resolution to conclusively model the sodium ion.

**Figure 6:**
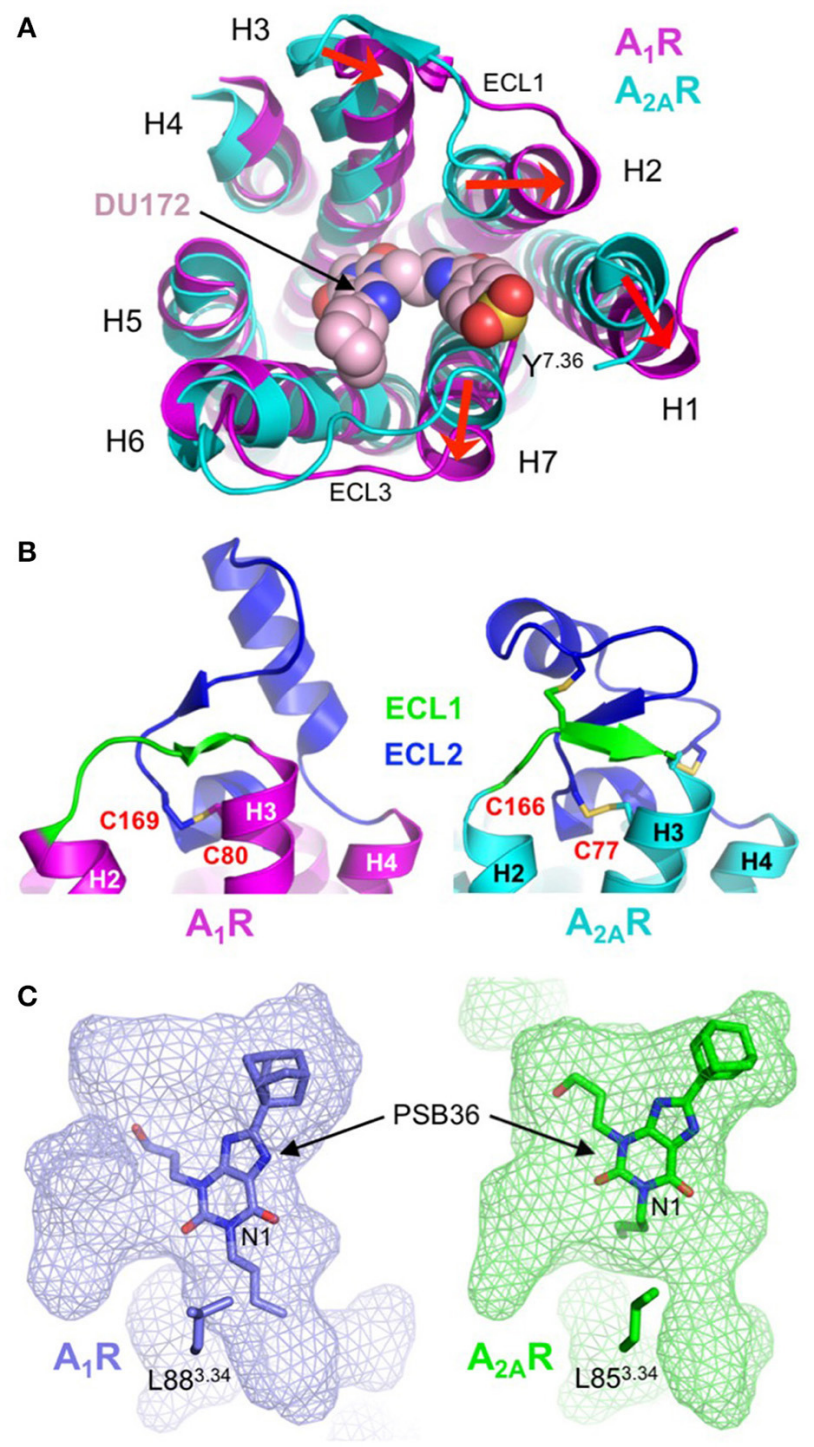
Structural diversity of the adenosine receptor family. **(A)** Extracellular view of the conformational differences between DU172-bound A_1_R (colored magenta; PDB: 5UEN) (Glukhova et al., 2017) and ZM241385-bound A_2A_R (colored cyan; PDB: 4EIY) (Liu et al., 2012). The extracellular ends of H1, H2 and H7 are displaced outwards by 5, 4, and 4 Å, respectively and H3 is displaced inwards by 4 Å (indicated by red arrows). The antagonist DU172 (shown as spheres) is covalently attached to Y271^7.36^ (shown as sticks) through a benzene sulfonate linkage. Note that for clarity, ECL2 has been omitted from the alignment. **(B)** Differential conformations of ECL1 (colored green) and ECL2 (colored blue) in A_1_R (colored magenta; PDB: 5UEN) (Glukhova et al., 2017) and A_2A_R (colored cyan; PDB: 4EIY) (Liu et al., 2012), disulphide bonds are shown as sticks. In A_1_R ECL2 adopts an extended conformation that is stabilized by a single disulphide bond (C80^3.25^-C169^ECL2^). In contrast, ECL2 from A_2A_R adopts a compact conformation that is stabilized by three disulphide bond, one of which (C77^3.25^-C166^ECL2^) is conserved in A_1_R. **(C)** Surface representation of the ligand-binding pocket (rendered as semi-transparent mesh) in A_1_R (colored blue; PDB: 5N2R) (Cheng et al., 2017) and A_2A_R (colored green; PDB: 5N2S) (Cheng et al., 2017), which highlights differences in both the topology of the orthosteric site and the binding orientation of PSB36 (shown as sticks). The butyl substituent at position N1 of the xanthine core of PSB36 fits into a channel between H3, H5, and H6 in A_1_R, this channel is constricted in A_2A_R due to the different orientation of L85^3.34^ (shown as sticks), which results in the ligand binding in a different orientation.

The most striking differences between A_1_R and A_2A_R are the conformational variations in extracellular ends of H1, H2, H3, and H7 and the orientation of ECL2. In the DU172-bound structure H3 is displaced inwards by 4 Å, and H1, H2, and H7 are displaced outwards by 5, 4, and 4 Å, respectively (Figure 6A). The outward movements in H1, H2, and H7 are required to accommodate the benzene sulfonate group of DU172, which is covalently linked to Y271^7.36^, and result in both the expansion of the orthosteric site and the formation of a secondary allosteric pocket (Glukhova et al., 2017). Direct comparison of A_1_R and A_2A_R bound to PSB36, which does not contain the benzene sulfonate substituent, also reveals a partial expansion of the orthosteric pocket (Cheng et al., 2017), indicating that this region of A_1_R is indeed more conformationally malleable than that of A_2A_R. Intriguingly, it has been suggested that the conformational rearrangements in H1, H2, and H3 in A_1_R may be a direct result of the different disulphide bond structure of ECL2 (Glukhova et al., 2017). In both A_1_R structures ECL2 adopts a similar conformation, which is different from that observed in any of the published A_2A_R structures (Figure 6B). The helical segment in ECL2 of A_1_R is extended by five residues and is positioned almost perpendicular to the transmembrane helices, compared to the near parallel arrangement in A_2A_R (Cheng et al., 2017; Glukhova et al., 2017). There is only a single disulphide bond in ECL2 of A_1_R compared to three within the same region of A_2A_R, which results in it adopting an extended conformation (Figure 6B). This appears to reduce conformational constraints on the extracellular ends of H2 and H3 allowing them to be displaced outwards, which in turn influences the positioning of H1. The displacement of H7 may also be linked to structural divergence in the extracellular loops, in this case a single amino acid truncation in ECL3 of A_1_R has been proposed to induce the outward tilt observed in the structures (Cheng et al., 2017; Glukhova et al., 2017).

What do these structures tell us about the molecular determinants of ligand-binding specificity in different AR subtypes? First, sequence differences in the binding pocket do not appear to be the main determinant of ligand-binding specificity in ARs. The orthosteric binding pocket of A_1_R and A_2A_R in the PSB36-bound structures differ by only four residues V62^2.57^, N70^2.65^, E170^ECL2^, and T270^7.35^ (corresponding to A59^2.57^, S67^2.65^, L170^ECL2^, and M270^7.35^ in A_2A_R), and of these, only T/M270^7.35^ form direct contacts with the ligand (Cheng et al., 2017). Mutagenesis studies have shown that residue 270^7.35^ is important in ligand-binding specificity, introducing the T270M mutation into A_1_R resulted in decreased binding affinity of A_1_R-specific ligands and increased binding affinity of A_2A_R-specific ligands, the reverse effect was observed when the M270T mutation was introduced into A_2A_R (Cheng et al., 2017; Glukhova et al., 2017). However, the positioning of this residue on the extracellular end of H7, at the perimeter of the binding pocket, suggests its main role is to act as a “gatekeeper” that regulates ligand access to the orthosteric site (Glukhova et al., 2017). Second, the topology of the binding pocket appears to play a central role in ligand-binding specificity. As described above the disulphide bond structure of ECL1 and ECL2 in A_1_R increases mobility in H1, H2 and H3, which causes an expansion of the binding pocket that is required to accommodate the benzene sulfonate group of the A_1_R-selective ligand DU172 (Figure 6A) (Glukhova et al., 2017). Binding pocket topology is also the predominant factor in the differential binding modes of PSB36 between A_1_R and A_2A_R (Figure 6C). PSB36 binds 2 Å deeper in the orthosteric pocket of A_1_R due to the presence of a channel between H3, H5 and H6 that can accommodate the butyl substituent at position N1 of the xanthine core (Figure 6C) (Cheng et al., 2017). This channel is created by a 2 Å displacement of L88^3.34^ in A_1_R that appears to be the result of an upstream proline residue (P86^3.31^), which is located outside the binding pocket, distorting the helix geometry in this region of H3. A proline at this position is unique to A_1_R and helps to explain why substitutions at position N1 of the xanthine core contribute to A_1_R selectivity (Cheng et al., 2017). Thus, the amino acid sequence both inside and outside the ligand-binding site, the extracellular loop structure and the topology of the binding pocket play interconnected roles in governing the ligand binding affinity and kinetics that are ultimately responsible for the functional selectivity of AR subtypes.

## Conclusion

A decade of research and innovation has culminated in the crystallization of more than 30 structures of human A_2A_R in complex with one inverse agonist, 11 antagonists and four agonists, as well as an engineered G protein. These structures represent the inactive, intermediate-active and active conformational states, and A_2A_R remains the only receptor for which this complete series of structures has been reported. Most of the structure were obtained using high affinity ligands, such as ZM241385, XAC, NECA, UK-432097, CGS21680. However, the application of conformational thermostabilization has also facilitated structure determination of the receptor in complex with lower affinity ligands, including the endogenous agonist adenosine and the natural plant-derived antagonists caffeine and theophylline. Structural characterization of the ligand-binding pocket of A_2A_R has provide novel insight into the binding modes of different classes of ligands; the ribose moiety has been identified as a key component of agonists that helps to stabilize the intermediate-active state before the receptor can adopt the fully active conformation upon G protein coupling. The chemical diversity of compounds co-crystallized with A_2A_R has also revealed how some ligands can exploit subsidiary binding sites on the extracellular surface of the receptor, as exemplified by the antagonists XAC, ZM241385, 8D1, T4G, and T4E and agonists CGS21680 and UK-432097.

High-resolution structures have not only provided a clear picture of the ligand-binding pocket, but have also highlighted the impact of receptor flexibility, notably in ECL2 and ECL3, on the mode and kinetics of ligand binding. Furthermore, the recently solved structures of A_1_R revealed that binding pocket topology and extracellular loop structure are two of the most important factors affecting the ligand binding specificity of different AR subtypes. These observations highlight the challenges of homology modeling GPCRs, since differential extracellular loop structures, global helix movements and changes in binding pocket topology are more difficult to model than amino acid substitutions within the orthosteric site. Thus, continued efforts to experimentally determine structures of all four AR subtypes in the three distinct activation states are essential to maximize the potential of structure based drug design for this family of receptors. Interestingly, despite the fact that all ARs are known to signal through G protein-independent pathways, no biased ligands have thus far been reported for A_2A_R (Verzijl and Ijzerman, 2011). Functional selectivity has now been observed in A_1_R, A_2B_R, and A_3_R (Gao et al., 2014; Baltos et al., 2016a,b), which suggests that biased ligands could also be developed for A_2A_R. However, it is also possible that subtle differences in the energy landscape and mechanism of A_2A_R activation may mean that biased signaling is less pronounced for this receptor. Therefore, at present A_2A_R is not an ideal model for studying functional selectivity, however structural insight from other GPCRs that have been co-crystallized with biased agonists, such as β_1_AR (Warne et al., 2012), may yet help to facilitate the design biased ligands for A_2A_R.

Finally, how will the wealth of high-quality structural data reported for A_2A_R shape the future of drug development for this receptor? Structural based design has already been used to develop novel A_2A_R antagonists, including a 1,2,4-triazine derivative that is a preclinical candidate for the treatment of Parkinson's disease (Congreve et al., 2012). Further application of this approach will likely result in the identification of additional novel compounds, both agonists and antagonists, and will also facilitate the derivatization and optimization of existing ligands. The identification of the subsidiary binding pocket on the extracellular surface of A_2A_R is an important step toward the design of allosteric modulators. Exploitation of this pocket has thus far been achieved using orthosteric ligands with large substituents that extend outside the orthosteric site, however in the future it may also be possible to target this region using purely allosteric ligands. Such compounds have the potential to modulate the properties of orthosteric ligands, and could thus be used to fine tune adenosine signaling through individual AR subtypes.

## Author contributions

All authors listed have made a substantial, direct, and intellectual contribution to the work, and approved it for publication.

### Conflict of interest statement

The authors declare that the research was conducted in the absence of any commercial or financial relationships that could be construed as a potential conflict of interest.
